# 6-Cyclo­hexyl­methyl-2-cyclo­hexyl­sul­fanyl-5-isopropyl­pyrimidin-4(3*H*)-one

**DOI:** 10.1107/S1600536808025592

**Published:** 2008-08-16

**Authors:** Chun-Sheng Zhang, Da-Xiong Li, De-Hua Zhang, Yan-Ping He, Cong Li

**Affiliations:** a School of Chemical Science and Technology, Yunnan University, Kunming 650091, People’s Republic of China; b School of Chemical Science and Technology, Key Laboratory of Medicinal Chemistry for Natural Resources, (Ministry of Education), Yunnan University, Kunming 650091, People’s Republic of China

## Abstract

The title compound, C_20_H_32_N_2_OS, was obtained during the course of our investigation on 2-alkylsulfanyl-6-benzyl-3,4-dihydropyrimidin-4(3*H*)-ones (S–DABOs) showing favourable anti-HIV-1 activity. Both cyclo­hexane rings adopt chair conformations. The angle at the methyl­ene C atom linking the pyrimidine and cyclo­hexane ring is 113.7 (3)°, which is in the range considered optimal for maximum activity of non-nucleoside reverse transcriptase inhibitors. Inter­molecular N—H⋯O hydrogen bonds link the mol­ecules into dimers and stabilize the crystal structure of the compound. In addition, an intra­molecular C—H⋯O hydrogen bond is observed.

## Related literature

For related literature, see: He *et al.* (2004 ▶); Ettorre *et al.* (1996 ▶, 1998 ▶); Rao *et al.* (2007 ▶).
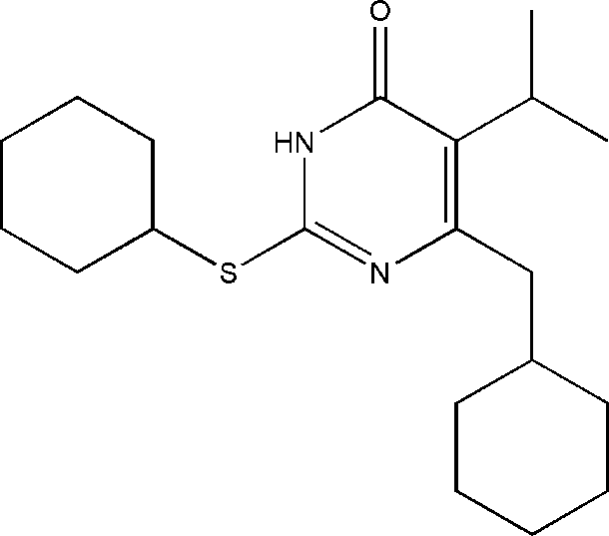


## Experimental

### 

#### Crystal data

C_20_H_32_N_2_OS
*M* *_r_* = 348.54
Triclinic, 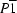

*a* = 9.9549 (16) Å
*b* = 10.9542 (17) Å
*c* = 12.1054 (19) Å
α = 63.250 (2)°
β = 69.195 (2)°
γ = 63.033 (2)°
*V* = 1031.5 (3) Å^3^
*Z* = 2
Mo *K*α radiation
μ = 0.17 mm^−1^
*T* = 298 (2) K
0.19 × 0.14 × 0.12 mm

#### Data collection

Bruker SMART CCD area-detector diffractometer
Absorption correction: multi-scan (**SADABS**; Bruker, 1998 ▶) *T*_min_ = 0.969, *T*_max_ = 0.980
9017 measured reflections
4697 independent reflections
2103 reflections with *I* > 2σ(*I*)
*R*_int_ = 0.046

#### Refinement

*R*[*F* ^2^ > 2σ(*F* ^2^)] = 0.065
*wR*(*F* ^2^) = 0.216
*S* = 0.88
4697 reflections
219 parameters
H-atom parameters constrained
Δρ_max_ = 0.17 e Å^−3^
Δρ_min_ = −0.20 e Å^−3^

### 

Data collection: *SMART* (Bruker, 1998 ▶); cell refinement: *SAINT* (Bruker, 1998 ▶); data reduction: *SAINT*; program(s) used to solve structure: *SHELXS97* (Sheldrick, 2008 ▶); program(s) used to refine structure: *SHELXL97* (Sheldrick, 2008 ▶); molecular graphics: *SHELXTL* (Sheldrick, 2008 ▶); software used to prepare material for publication: *SHELXTL*.

## Supplementary Material

Crystal structure: contains datablocks I, global. DOI: 10.1107/S1600536808025592/wn2273sup1.cif

Structure factors: contains datablocks I. DOI: 10.1107/S1600536808025592/wn2273Isup2.hkl

Additional supplementary materials:  crystallographic information; 3D view; checkCIF report

## Figures and Tables

**Table 1:** Hydrogen-bond geometry (Å, °)

*D*—H⋯*A*	*D*—H	H⋯*A*	*D*⋯*A*	*D*—H⋯*A*
N2—H2⋯O1^i^	0.86	1.91	2.761 (4)	170
C11—H11*C*⋯O1	0.96	2.53	3.115 (5)	119

Symmetry code: (i) 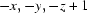.

**Fig. 1.**
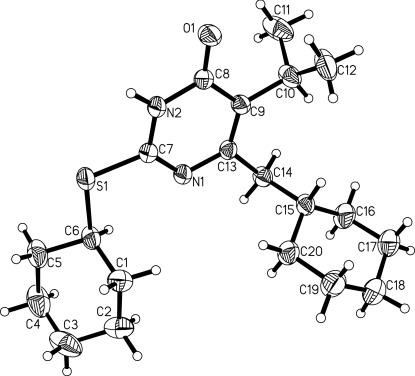
The molecular structure of the title compound, showing the atom labelling scheme and 30% probability displacement ellipsoids.

**Fig. 2.**
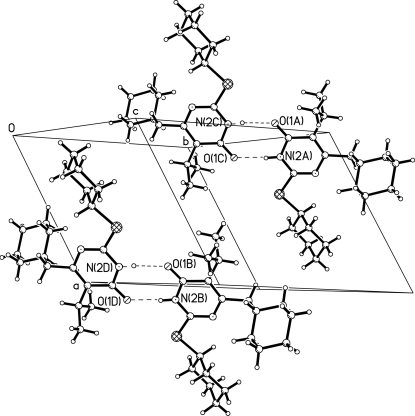
The crystal packing of title compound showing the intermolecular hydrogen bonding (dashed lines)

## supplementary crystallographic information

### Comment

As part of our ongoing investigation of S-DABO analogues which are a potent family of non-nucleoside reverse transcriptase inhibitors (NNRTIs), the title compound was synthesized as a novel inhibitor and shows favourable anti-HIV-1 activity.

The molecular structure is shown in Fig. 1. Both cyclohexane rings adopt the lowest energy chair conformation. C13—C14—C5 is 113.7 (3)°, which is in the range considered optimal for maximum activity of NNRTIs, *viz*. 110°–115° (Ettorre *et al.*, 1996).

A comparision of the crystal structure of the title compound with some reported S-DABOs show that their spatial arrangement is similar (Ettorre *et al.*, 1998; Rao *et al.*, 2007). Although these molecules assume a similar conformation, they show differences in their activities. Thus, futher structural investigations are needed.

Intermolecular N—H···O hydrogen bonds link the molecules into dimers and stabilize the crystal structure of the compound. In addition, an intramolecular C—H···O hydrogen bond is observed.

### Experimental

With 2-cyclohexylacetonitrile as the starting material, the title compound was synthesized according to the procedure of He *et al.* (2004). Single crystals were obtained from a mixture of ethyl acetate and petroleum ether by slow evaporation at room temperature.

### Refinement

Methyl H atoms were placed in calculated positions with C—H = 0.96 Å and the torsion angle was refined to fit the electron density; *U*_iso_(H) = 1.5*U*_eq_(C). Other H atoms were placed in calculated positions with C—H = 0.97–0.98 Å and N—H = 0.86 Å, and refined in riding mode; *U*_iso_(H) = 1.2*U*_eq_(C, N).

### Crystal data

**Table:**

C_20_H_32_N_2_OS	*Z* = 2
*M**_r_* = 348.54	*F*_000_ = 380
Triclinic, *P*1	*D*_x_ = 1.122 Mg m^−^^3^
*a* = 9.9549 (16) Å	Mo *K*α radiation λ = 0.71073 Å
*b* = 10.9542 (17) Å	Cell parameters from 4697 reflections
*c* = 12.1054 (19) Å	θ = 1.9–28.3º
α = 63.250 (2)º	µ = 0.17 mm^−^^1^
β = 69.195 (2)º	*T* = 298 (2) K
γ = 63.033 (2)º	Block, colourless
*V* = 1031.5 (3) Å^3^	0.19 × 0.14 × 0.12 mm

### Data collection

**Table:**

Bruker SMART CCD area-detector diffractometer	4697 independent reflections
Radiation source: fine-focus sealed tube	2103 reflections with *I* > 2σ(*I*)
Monochromator: graphite	*R*_int_ = 0.047
*T* = 298(2) K	θ_max_ = 28.3º
φ and ω scans	θ_min_ = 1.9º
Absorption correction: multi-scan(*SADABS*; Bruker, 1998)	*h* = −13→13
*T*_min_ = 0.969, *T*_max_ = 0.980	*k* = −13→14
9017 measured reflections	*l* = −16→15

### Refinement

**Table:**

Refinement on *F*^2^	Secondary atom site location: difference Fourier map
Least-squares matrix: full	Hydrogen site location: inferred from neighbouring sites
*R*[*F*^2^ > 2σ(*F*^2^)] = 0.065	H-atom parameters constrained
*wR*(*F*^2^) = 0.216	*w* = 1/[σ^2^(*F*_o_^2^) + (0.1*P*)^2^ + 0.3399*P*] where *P* = (*F*_o_^2^ + 2*F*_c_^2^)/3
*S* = 0.88	(Δ/σ)_max_ < 0.001
4697 reflections	Δρ_max_ = 0.18 e Å^−^^3^
219 parameters	Δρ_min_ = −0.20 e Å^−^^3^
Primary atom site location: structure-invariant direct methods	Extinction correction: none

### Special details

**Table:**

Geometry. All e.s.d.'s (except the e.s.d. in the dihedral angle between two l.s. planes)are estimated using the full covariance matrix. The cell e.s.d.'s are takeninto account individually in the estimation of e.s.d.'s in distances, anglesand torsion angles; correlations between e.s.d.'s in cell parameters are onlyused when they are defined by crystal symmetry. An approximate (isotropic)treatment of cell e.s.d.'s is used for estimating e.s.d.'s involving l.s. planes.
Refinement. Refinement of *F*^2^ against ALL reflections. The weighted *R*-factor *wR* and goodness of fit *S* are based on *F*^2^, conventional *R*-factors *R* are based on *F*, with *F* set to zero for negative *F*^2^. The threshold expression of *F*^2^ > σ(*F*^2^) is used only for calculating *R*-factors(gt) *etc*, and is not relevant to the choice of reflections for refinement. *R*-factors based on *F*^2^ are statistically about twice as large as those based on *F*, and *R*-factors based on ALL data will be even larger.

### Fractional atomic coordinates and isotropic or equivalent isotropic displacement parameters (Å^2^)

**Table:**

	*x*	*y*	*z*	*U*_iso_*/*U*_eq_	
S1	0.33860 (11)	0.15552 (12)	0.30395 (9)	0.0638 (3)	
O1	−0.1114 (3)	0.0731 (3)	0.6134 (2)	0.0669 (8)	
N1	0.1985 (3)	0.2729 (3)	0.4863 (2)	0.0473 (7)	
N2	0.0978 (3)	0.1230 (3)	0.4780 (2)	0.0514 (7)	
H2	0.1069	0.0683	0.4405	0.062*	
C8	−0.0217 (4)	0.1378 (4)	0.5792 (3)	0.0492 (8)	
C7	0.2013 (4)	0.1900 (4)	0.4349 (3)	0.0465 (8)	
C13	0.0816 (3)	0.2939 (3)	0.5876 (3)	0.0439 (8)	
C9	−0.0283 (4)	0.2320 (4)	0.6356 (3)	0.0492 (8)	
C10	−0.1620 (4)	0.2617 (4)	0.7421 (4)	0.0686 (11)	
H10	−0.1477	0.3255	0.7702	0.082*	
C14	0.0877 (4)	0.3902 (4)	0.6429 (3)	0.0522 (9)	
H14A	−0.0149	0.4592	0.6585	0.063*	
H14B	0.1521	0.4456	0.5817	0.063*	
C6	0.4652 (4)	0.2481 (4)	0.2780 (3)	0.0551 (9)	
H6	0.4021	0.3453	0.2833	0.066*	
C15	0.1495 (4)	0.3055 (4)	0.7659 (3)	0.0531 (9)	
H15	0.0998	0.2329	0.8188	0.064*	
C5	0.5566 (5)	0.2656 (5)	0.1449 (4)	0.0801 (13)	
H5A	0.4871	0.3252	0.0857	0.096*	
H5B	0.6106	0.1706	0.1356	0.096*	
C1	0.5698 (4)	0.1675 (4)	0.3730 (4)	0.0675 (11)	
H1A	0.6276	0.0684	0.3734	0.081*	
H1B	0.5087	0.1628	0.4566	0.081*	
C20	0.3203 (4)	0.2241 (4)	0.7442 (4)	0.0671 (11)	
H20A	0.3720	0.2930	0.6876	0.081*	
H20B	0.3431	0.1559	0.7039	0.081*	
C16	0.1092 (5)	0.4041 (5)	0.8375 (4)	0.0779 (12)	
H16A	0.1537	0.4795	0.7857	0.094*	
H16B	−0.0014	0.4514	0.8545	0.094*	
C18	0.3361 (5)	0.2395 (5)	0.9399 (4)	0.0860 (13)	
H18A	0.3673	0.1805	1.0210	0.103*	
H18B	0.3898	0.3084	0.8947	0.103*	
C2	0.6803 (5)	0.2449 (5)	0.3405 (4)	0.0806 (13)	
H2A	0.6225	0.3418	0.3455	0.097*	
H2B	0.7477	0.1910	0.4013	0.097*	
C4	0.6718 (6)	0.3368 (6)	0.1148 (4)	0.1012 (17)	
H4A	0.7343	0.3389	0.0320	0.121*	
H4B	0.6168	0.4369	0.1124	0.121*	
C17	0.1662 (5)	0.3218 (6)	0.9600 (4)	0.0909 (15)	
H17A	0.1430	0.3900	1.0003	0.109*	
H17B	0.1131	0.2538	1.0158	0.109*	
C19	0.3812 (5)	0.1413 (5)	0.8657 (4)	0.0842 (13)	
H19A	0.4922	0.0985	0.8466	0.101*	
H19B	0.3415	0.0624	0.9170	0.101*	
C3	0.7749 (5)	0.2569 (6)	0.2104 (6)	0.1060 (18)	
H3A	0.8383	0.1600	0.2068	0.127*	
H3B	0.8420	0.3090	0.1908	0.127*	
C12	−0.1652 (6)	0.1229 (6)	0.8561 (4)	0.0948 (15)	
H12A	−0.1853	0.0600	0.8335	0.142*	
H12B	−0.2446	0.1483	0.9239	0.142*	
H12C	−0.0679	0.0732	0.8823	0.142*	
C11	−0.3132 (4)	0.3452 (5)	0.6950 (5)	0.0978 (16)	
H11A	−0.3070	0.4320	0.6245	0.147*	
H11B	−0.3949	0.3714	0.7613	0.147*	
H11C	−0.3330	0.2846	0.6694	0.147*	

### Atomic displacement parameters (Å^2^)

**Table:**

	*U*^11^	*U*^22^	*U*^33^	*U*^12^	*U*^13^	*U*^23^
S1	0.0637 (6)	0.0911 (8)	0.0556 (6)	−0.0395 (6)	0.0099 (4)	−0.0446 (6)
O1	0.0614 (16)	0.088 (2)	0.0772 (18)	−0.0426 (15)	0.0112 (13)	−0.0516 (16)
N1	0.0487 (16)	0.0526 (17)	0.0457 (16)	−0.0201 (14)	−0.0045 (12)	−0.0222 (14)
N2	0.0519 (16)	0.0645 (19)	0.0499 (16)	−0.0248 (15)	0.0003 (13)	−0.0329 (15)
C8	0.0451 (19)	0.059 (2)	0.051 (2)	−0.0177 (17)	−0.0052 (15)	−0.0282 (17)
C7	0.0478 (19)	0.052 (2)	0.0429 (18)	−0.0194 (16)	−0.0072 (14)	−0.0181 (16)
C13	0.0449 (18)	0.046 (2)	0.0426 (18)	−0.0123 (15)	−0.0075 (14)	−0.0211 (16)
C9	0.0432 (18)	0.058 (2)	0.053 (2)	−0.0192 (16)	−0.0007 (15)	−0.0295 (17)
C10	0.063 (2)	0.082 (3)	0.080 (3)	−0.037 (2)	0.018 (2)	−0.055 (2)
C14	0.052 (2)	0.053 (2)	0.057 (2)	−0.0199 (17)	−0.0023 (16)	−0.0273 (18)
C6	0.061 (2)	0.054 (2)	0.052 (2)	−0.0245 (18)	0.0040 (17)	−0.0273 (18)
C15	0.060 (2)	0.062 (2)	0.050 (2)	−0.0301 (18)	0.0006 (16)	−0.0299 (18)
C5	0.099 (3)	0.096 (3)	0.058 (2)	−0.060 (3)	0.017 (2)	−0.036 (2)
C1	0.058 (2)	0.069 (3)	0.072 (3)	−0.022 (2)	−0.015 (2)	−0.020 (2)
C20	0.064 (2)	0.076 (3)	0.066 (2)	−0.013 (2)	−0.0122 (19)	−0.040 (2)
C16	0.082 (3)	0.091 (3)	0.080 (3)	−0.021 (2)	−0.011 (2)	−0.060 (3)
C18	0.093 (3)	0.109 (4)	0.074 (3)	−0.038 (3)	−0.018 (2)	−0.043 (3)
C2	0.062 (3)	0.075 (3)	0.113 (4)	−0.023 (2)	−0.024 (3)	−0.035 (3)
C4	0.130 (4)	0.115 (4)	0.079 (3)	−0.089 (4)	0.035 (3)	−0.045 (3)
C17	0.091 (3)	0.128 (4)	0.080 (3)	−0.033 (3)	−0.005 (2)	−0.072 (3)
C19	0.080 (3)	0.094 (3)	0.082 (3)	−0.012 (2)	−0.026 (2)	−0.043 (3)
C3	0.067 (3)	0.101 (4)	0.168 (6)	−0.042 (3)	0.016 (3)	−0.078 (4)
C12	0.112 (4)	0.116 (4)	0.066 (3)	−0.066 (3)	0.029 (3)	−0.048 (3)
C11	0.053 (3)	0.095 (4)	0.133 (4)	−0.021 (2)	0.017 (3)	−0.062 (3)

### Geometric parameters (Å, °)

**Table:**

S1—C7	1.748 (3)	C1—H1B	0.9700
S1—C6	1.817 (3)	C20—C19	1.513 (5)
O1—C8	1.233 (4)	C20—H20A	0.9700
N1—C7	1.299 (4)	C20—H20B	0.9700
N1—C13	1.381 (4)	C16—C17	1.505 (6)
N2—C7	1.353 (4)	C16—H16A	0.9700
N2—C8	1.383 (4)	C16—H16B	0.9700
N2—H2	0.8600	C18—C17	1.502 (6)
C8—C9	1.441 (4)	C18—C19	1.520 (6)
C13—C9	1.364 (4)	C18—H18A	0.9700
C13—C14	1.512 (4)	C18—H18B	0.9700
C9—C10	1.518 (4)	C2—C3	1.503 (6)
C10—C11	1.523 (6)	C2—H2A	0.9700
C10—C12	1.531 (6)	C2—H2B	0.9700
C10—H10	0.9800	C4—C3	1.509 (7)
C14—C15	1.531 (5)	C4—H4A	0.9700
C14—H14A	0.9700	C4—H4B	0.9700
C14—H14B	0.9700	C17—H17A	0.9700
C6—C1	1.514 (5)	C17—H17B	0.9700
C6—C5	1.516 (5)	C19—H19A	0.9700
C6—H6	0.9800	C19—H19B	0.9700
C15—C20	1.508 (5)	C3—H3A	0.9700
C15—C16	1.511 (5)	C3—H3B	0.9700
C15—H15	0.9800	C12—H12A	0.9600
C5—C4	1.521 (6)	C12—H12B	0.9600
C5—H5A	0.9700	C12—H12C	0.9600
C5—H5B	0.9700	C11—H11A	0.9600
C1—C2	1.523 (5)	C11—H11B	0.9600
C1—H1A	0.9700	C11—H11C	0.9600
			
C7—S1—C6	103.08 (16)	C19—C20—H20B	109.2
C7—N1—C13	117.1 (3)	H20A—C20—H20B	107.9
C7—N2—C8	123.4 (3)	C17—C16—C15	112.2 (4)
C7—N2—H2	118.3	C17—C16—H16A	109.2
C8—N2—H2	118.3	C15—C16—H16A	109.2
O1—C8—N2	119.8 (3)	C17—C16—H16B	109.2
O1—C8—C9	125.8 (3)	C15—C16—H16B	109.2
N2—C8—C9	114.4 (3)	H16A—C16—H16B	107.9
N1—C7—N2	122.9 (3)	C17—C18—C19	111.7 (4)
N1—C7—S1	123.0 (3)	C17—C18—H18A	109.3
N2—C7—S1	114.1 (2)	C19—C18—H18A	109.3
C9—C13—N1	123.6 (3)	C17—C18—H18B	109.3
C9—C13—C14	122.5 (3)	C19—C18—H18B	109.3
N1—C13—C14	113.8 (3)	H18A—C18—H18B	107.9
C13—C9—C8	118.6 (3)	C3—C2—C1	111.1 (4)
C13—C9—C10	123.7 (3)	C3—C2—H2A	109.4
C8—C9—C10	117.7 (3)	C1—C2—H2A	109.4
C9—C10—C11	110.7 (3)	C3—C2—H2B	109.4
C9—C10—C12	112.6 (3)	C1—C2—H2B	109.4
C11—C10—C12	112.1 (3)	H2A—C2—H2B	108.0
C9—C10—H10	107.0	C3—C4—C5	112.2 (4)
C11—C10—H10	107.0	C3—C4—H4A	109.2
C12—C10—H10	107.0	C5—C4—H4A	109.2
C13—C14—C15	113.7 (3)	C3—C4—H4B	109.2
C13—C14—H14A	108.8	C5—C4—H4B	109.2
C15—C14—H14A	108.8	H4A—C4—H4B	107.9
C13—C14—H14B	108.8	C18—C17—C16	111.4 (3)
C15—C14—H14B	108.8	C18—C17—H17A	109.3
H14A—C14—H14B	107.7	C16—C17—H17A	109.3
C1—C6—C5	111.3 (3)	C18—C17—H17B	109.3
C1—C6—S1	113.1 (3)	C16—C17—H17B	109.3
C5—C6—S1	106.7 (2)	H17A—C17—H17B	108.0
C1—C6—H6	108.5	C20—C19—C18	111.9 (4)
C5—C6—H6	108.5	C20—C19—H19A	109.2
S1—C6—H6	108.5	C18—C19—H19A	109.2
C20—C15—C16	110.4 (3)	C20—C19—H19B	109.2
C20—C15—C14	112.2 (3)	C18—C19—H19B	109.2
C16—C15—C14	111.6 (3)	H19A—C19—H19B	107.9
C20—C15—H15	107.5	C2—C3—C4	110.2 (4)
C16—C15—H15	107.5	C2—C3—H3A	109.6
C14—C15—H15	107.5	C4—C3—H3A	109.6
C6—C5—C4	111.2 (3)	C2—C3—H3B	109.6
C6—C5—H5A	109.4	C4—C3—H3B	109.6
C4—C5—H5A	109.4	H3A—C3—H3B	108.1
C6—C5—H5B	109.4	C10—C12—H12A	109.5
C4—C5—H5B	109.4	C10—C12—H12B	109.5
H5A—C5—H5B	108.0	H12A—C12—H12B	109.5
C6—C1—C2	110.4 (3)	C10—C12—H12C	109.5
C6—C1—H1A	109.6	H12A—C12—H12C	109.5
C2—C1—H1A	109.6	H12B—C12—H12C	109.5
C6—C1—H1B	109.6	C10—C11—H11A	109.5
C2—C1—H1B	109.6	C10—C11—H11B	109.5
H1A—C1—H1B	108.1	H11A—C11—H11B	109.5
C15—C20—C19	112.1 (3)	C10—C11—H11C	109.5
C15—C20—H20A	109.2	H11A—C11—H11C	109.5
C19—C20—H20A	109.2	H11B—C11—H11C	109.5
C15—C20—H20B	109.2		
			
C7—N2—C8—O1	−179.8 (3)	C9—C13—C14—C15	75.8 (4)
C7—N2—C8—C9	0.3 (5)	N1—C13—C14—C15	−103.1 (3)
C13—N1—C7—N2	−1.4 (5)	C7—S1—C6—C1	74.8 (3)
C13—N1—C7—S1	178.5 (2)	C7—S1—C6—C5	−162.5 (3)
C8—N2—C7—N1	1.0 (5)	C13—C14—C15—C20	73.0 (4)
C8—N2—C7—S1	−178.9 (2)	C13—C14—C15—C16	−162.6 (3)
C6—S1—C7—N1	4.1 (3)	C1—C6—C5—C4	−53.9 (5)
C6—S1—C7—N2	−176.0 (2)	S1—C6—C5—C4	−177.7 (3)
C7—N1—C13—C9	0.4 (5)	C5—C6—C1—C2	55.9 (4)
C7—N1—C13—C14	179.3 (3)	S1—C6—C1—C2	176.0 (3)
N1—C13—C9—C8	0.9 (5)	C16—C15—C20—C19	54.5 (5)
C14—C13—C9—C8	−177.9 (3)	C14—C15—C20—C19	179.6 (3)
N1—C13—C9—C10	−176.4 (3)	C20—C15—C16—C17	−55.8 (5)
C14—C13—C9—C10	4.8 (5)	C14—C15—C16—C17	178.7 (3)
O1—C8—C9—C13	179.0 (3)	C6—C1—C2—C3	−58.1 (5)
N2—C8—C9—C13	−1.2 (5)	C6—C5—C4—C3	54.0 (5)
O1—C8—C9—C10	−3.6 (5)	C19—C18—C17—C16	−53.6 (5)
N2—C8—C9—C10	176.2 (3)	C15—C16—C17—C18	55.8 (5)
C13—C9—C10—C11	112.6 (4)	C15—C20—C19—C18	−53.5 (5)
C8—C9—C10—C11	−64.7 (4)	C17—C18—C19—C20	52.6 (5)
C13—C9—C10—C12	−121.1 (4)	C1—C2—C3—C4	57.6 (5)
C8—C9—C10—C12	61.7 (5)	C5—C4—C3—C2	−55.7 (5)

### Hydrogen-bond geometry (Å, °)

**Table:**

*D*—H···*A*	*D*—H	H···*A*	*D*···*A*	*D*—H···*A*
N2—H2···O1^i^	0.86	1.91	2.761 (4)	170
C11—H11C···O1	0.96	2.53	3.115 (5)	119

Symmetry codes: (i) −*x*, −*y*, −*z*+1.
